# Long-Term Comorbid Neuropsychiatric Sequelae of Hypoxia at Birth

**DOI:** 10.7759/cureus.12687

**Published:** 2021-01-13

**Authors:** Christina Mercogliano, Karuna Poddar

**Affiliations:** 1 Psychiatry, Thomas Jefferson University Hospital, Philadelphia, USA

**Keywords:** perinatal hypoxia, asphyxia, neuropsychiatric, mood disorder, adhd, aggression

## Abstract

Perinatal hypoxia due to obstetric complications has been known to cause neurodevelopmental impairments in infants and children. The severity of the impairments and recovery depends on the degree of hypoxia. There have been some studies which focuses on understanding the effects of perinatal hypoxia on cognitive and behavioral functioning like attention-deficit/hyperactivity disorder (ADHD), autism spectrum disorder (ASD), learning disorders, or aggression. Although the studies have investigated the effects in children, there are very few studies done to explore perinatal hypoxia, causing any neuropsychiatric outcomes in adults.

This is a case of a 38-year-old man who presented to psychiatry as a referral for depression by neurology. He saw neurology for intractable migraine resistant to all treatment for the last year. The brain imaging was read as normal with minor small vascular changes. During our assessment, he reported depression and passive suicidal ideation, which began since he was diagnosed with migraines. His developmental history was significant for perinatal asphyxia and learning difficulties. Growing up, he reported severe irritability, impulsivity, and risk-taking behaviors but became stable when he was in his late twenties. His past psychiatric management was unclear. He was seeing an outpatient therapist when he visited our clinic. We diagnosed him with an unspecified mood disorder, tried prozac, and then gabapentin with some effect. Before we could explore further medication trials with topamax, his care had to be transferred to other psychiatrists, and we could not obtain further details of his outcome.

Based on our case, we concluded there is a need for further research focused on the effects of perinatal hypoxia on certain brain areas as a cause of neuropsychiatric symptoms in adults.

## Introduction

Perinatal hypoxia and asphyxia due to obstetric complications are followed through the neonatal period to cause various neonatal encephalopathies, seizure disorders, or cerebral palsy [1]. Many children with moderate-to-severe encephalopathy have been known to have intellectual disabilities and severe learning difficulties [2]. We performed a thorough literature search to identify any long-term effects due to perinatal hypoxia on behavior or psychiatric deficits. There are few studies that have identified neurodevelopmental disorders like attention-deficit/hyperactivity disorder (ADHD) or autism spectrum disorder (ASD) [2]. There are even fewer studies identifying any functional behavioral deficits or cognitive effects in school-aged children [3]. Perinatal hypoxia due to birth complications has been associated with a higher risk of behavioral disturbance in childhood, but it is challenging to follow the behavioral disturbance in adulthood. ADHD has been studied in response to data from a rat model of neonatal hypoxia-ischemia, revealing inattention, impulsivity, and dopamine disturbances in the prefrontal cortex [4]. Animals subjected to hypoxia-ischemia were found to have impaired executive function associated with tissue atrophy and dopaminergic disturbance in the prefrontal cortex [5]. There has been discussion regarding hypoxia in perinatal infants and the long-term effects of behavioral problems such as aggression, impulsivity, ADHD, and ASD. We were able to find a few studies identifying the neuropsychiatric effects of hypoxia in adults. One study was done regarding the effects of hypoxia on the amygdala and hippocampus; it affected their sizes and determines the outcomes of bipolar disorder or schizophrenia [6]. Most studies have focused on detecting major developmental abnormalities at a young age, so little is known about the effects in the long run. Descriptions of long-term sequelae of perinatal asphyxia in adults are minimal in the literature, though changes in cognitive function and social behaviors have been described in animals [7].

## Case presentation

This is a case of a 38-year-old man who presented to psychiatry as a referral for depression by neurology. He had been followed by the neurology department for intractable migraine resistant to all treatment for the last year. His past medical history was also significant for obstructive sleep apnea and obesity. The patient arrived at our clinic with dark sunglasses and was accompanied by his mother. He reported that he had had headaches for the last year with no relief. He denied any history of prior headaches. Imaging was performed to look for an organic cause of the headache and rule out structural deformities. A CT of the head without contrast revealed a nonspecific deep subcutaneous soft tissue mass overlying the left occipital bone, measuring approximately 2 cm at the basal diameter. An MRI of the brain without contrast showed small vessel changes. Under the care of neurology, the patient tried sumatriptan, rizatriptan, topiramate, propranolol, nortriptyline, trazodone, and valproic acid and had recently undergone Botox treatment with minimal relief. He rated the pain an eight out of ten in the moment and a ten out of ten at its worst. The pain was frontal and located over his left eye. This was debilitating him to an extent where he had to resign from his job last year as a truck driver; since then he had been homebound. He preferred to stay in his darkened room all day. He stated that people talking, bright lights, and outside noise made his pain worse; therefore, he had no interest in going out and seeing people. He reported sleep lasted only three to four hours a night, but since he received his sleep apnea machine a couple of weeks ago, his sleep increased to four to seven hours a night. However, his sleep continued to be disturbed, with ongoing headaches, and he lacked energy when he woke up from sleep. He also developed poor appetite, hopelessness, depressed mood, and passive suicidal ideation. These feelings had gradually worsened in the past year. He denied suicide attempts or inpatient psychiatric admissions in the last year. He was seeing an outpatient therapist.

His developmental history was significant for perinatal asphyxia. Collateral information was collected from his mother, who described complications at birth, stating that the “cord was wrapped around his neck, and he came out blue.” Growing up, he reported severe irritability, impulsivity, and risk-taking behaviors. According to the mother, when she and her husband got divorced, the patient reacted impulsively by holding a knife to his own throat. He also, at the peak of his impulsivity, tried to hang himself on the neighbor’s tree when he was 10 years old. She states the patient never physically harmed anyone but did push his sister up against a car once for driving too quickly and caused his mother to hit her head. The patient was not evaluated by a child psychiatrist at that time as far as we know.

He admitted to homicidal ideation in the past and road rage without physical altercations. The patient also reported having learning difficulties in school, being unable to concentrate, and thus unable to complete his education. He reported that his behavioral issues and mood symptoms suddenly stabilized after he received a warning for his ill-conduct at the age of 23 from a judge. According to the patient, he did well from the age of 23; he led a stable lifestyle as a truck driver, denied road rage, and denied illicit substance use. He denied any hypomanic or depressive mood symptoms until last year when the headaches began. He did not receive any psychiatric care during that period. However, since the headaches began, he was seeing an outpatient therapist who diagnosed him with bipolar disorder. He was prescribed valproic acid 1500 mg daily for his mood symptoms and headaches by neurology.

Our clinic diagnosed him with an unspecified mood disorder and initially tried prozac for his depression and impulsivity symptoms. The patient did not observe any change in mood symptoms but felt his headaches were worsening on prozac. Considering his history of perinatal asphyxia, small vessel changes seen on MRI, and constant headaches along with mood symptoms, we started a trial of gabapentin. We communicated our concerns to neurology regarding his headaches as likely being secondary to cerebral vasculitis; however, the workup was pending. On gabapentin, the patient had observed his headaches and his mood symptoms to be better; however, he developed extensive bipedal edema. He was unable to walk, so the medication was discontinued. As we continued to explore further options, his mother later reported a history of migraine and mood symptoms in herself that had responded well to topamax. We always collaborated care with a neurologist and discussed the possibility of a trial of topamax. However, before it could be started, the patient’s care had to be transferred to another psychiatrist, and we could not obtain further details concerning his outcome.

## Discussion

The patient in our case displayed signs of impulsivity and irritability as a young child. These behaviors are often observed in children with ADHD or with disruptive mood dysregulation disorders (DMDD), both of which are associated with limited development of executive functions [8]. There have been studies that show that language learning and mood dysregulation can coexist in children diagnosed with DMDD, and our patient had an underlying history of a learning disorder. There may be a correlation from the perinatal asphyxia to his executive function and decision-making capabilities, similar to the findings in the animal studies previously discussed and cited. Perinatal events are linked with neurodevelopmental disorders like ADHD or ASD [9], which helped us understand that our patient had a higher risk of developing a neurodevelopmental disorder such as ADHD. It is unclear if the patient had DMDD as well; however, a pre-existing diagnosis of ADHD and mood disorders like DMDD likely predisposes an adult diagnosis of bipolar disorder or major depressive disorder [10]. It is also observed that impulsivity could be a result of a number of effects of perinatal asphyxia [11]. The ischemic and anoxic brain damage sustained during perinatal asphyxia could be directly linked to impulsivity and poor executive function such as in patients with a traumatic brain injury [12]. When he was younger, the patient's self-injurious behaviors may have been signs of impulsivity in the context of ADHD. The seriousness of the externalizing behaviors could also be a result of his mood dysregulation.

**Figure 1:**
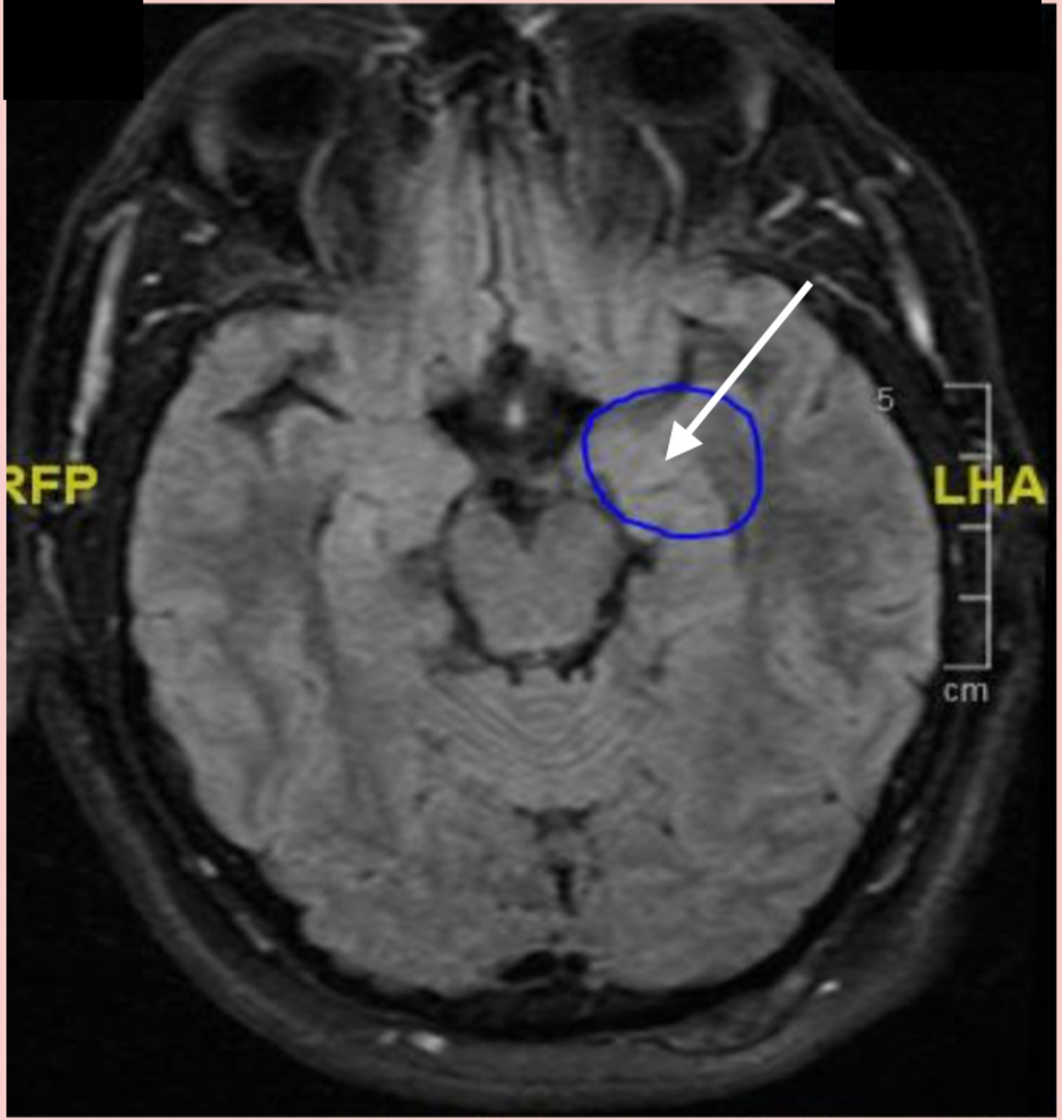
MRI brain showing amygdala enlargement.

Perinatal asphyxia is also known to cause hypoxic-induced encephalopathy, which can lead to seizures, based on citations for hypoxia-induced encephalopathy. There is some association of temporal lobe epilepsy with amygdala enlargement [13], and patients with psychotic bipolar disorder showed distinct associations between perinatal asphyxia and smaller left amygdala volume [6]. Abnormal amygdala size can lead to emotional dysregulation and stronger association with suicide, impulsivity, and aggressiveness; this could explain some of the patient’s symptoms such as the suicide attempt and road rage (Figure 1). As far as we know about the case, there were no concerns about any seizure disorder. In addition, the patient’s MRI revealed small vessel disease that could underlie an undiagnosed vasculitis-like condition causing him chronic headaches [14]. The sudden stabilization of the symptoms in his young adulthood would require some exploration. However, according to the patient’s history, there appeared to be a complete resolution of his impulsivity for the next 10 years of his life. Also in his case, it seems unclear if there could be a correlation with the intracerebral small vessel disease, migraine diagnosis, and the recurring onset of mood dysregulation in his middle adulthood [15-16]. 

It is likely our patient had psychiatric sequelae of ADHD with mood dysregulation and some underlying neurological sequelae leading to headaches as a result of his perinatal asphyxia; these were our working diagnoses. He also showed a positive response to gabapentin though it had to be discontinued due to the pedal edema identified as a side effect. It would have been interesting to know if he would respond to topamax as noted by the good response in a family member with similar medical symptoms.

## Conclusions

A missed diagnosis can lead to mistreatment and misunderstanding of the patient’s needs. However, it is a challenge to appropriately recognize and treat long-term perinatal asphyxia sequelae in an adult patient. There is, in fact, currently no reliable measure of brain function, brain oxygenation, or cerebral blood flow during the prenatal period or the intrapartum period. We recognize that to perform a longitudinal study is an arduous task, so it would be a challenge to demonstrate the correlation between injury at birth and behaviors in the adult years. It is certainly something to consider when a subject appears to have both psychiatric and neurological deficits with a history of plausible perinatal asphyxia. A better understanding of the developmental changes in brain maturity can prevent life-altering behaviors and physical ailments.

Based on our case study, we conclude that a missed diagnosis may lead to a delay in appropriate treatment; and that developmental changes in brain maturity may lead to life-long neurological and psychiatric conditions.
